# Weekend and off-hour effects on the incidence of cerebral palsy: contribution of consolidated perinatal care

**DOI:** 10.1186/s12199-020-00889-y

**Published:** 2020-09-10

**Authors:** Satoshi Toyokawa, Junichi Hasegawa, Tsuyomu Ikenoue, Yuri Asano, Emi Jojima, Shoji Satoh, Tomoaki Ikeda, Kiyotake Ichizuka, Satoru Takeda, Nanako Tamiya, Akihito Nakai, Keiya Fujimori, Tsugio Maeda, Hideaki Masuzaki, Hideaki Suzuki, Shigeru Ueda

**Affiliations:** 1 Department of Public Health, Graduate School of Medicine, The University of Tokyo, 7-3-1 Hongo, Bunkyo-ku, Tokyo, 113-0033 Japan; 2 Department of the Japan Obstetric Compensation System for Cerebral Palsy, Japan Council for Quality Health Care, Tokyo, Japan; 3 Department of Obstetrics and Gynecology, St. Marianna University School of Medicine, Kanagawa, Japan; 4 University of Miyazaki, Miyazaki, Japan; 5 Maternal and Perinatal Care Center, Oita Prefectural Hospital, Oita, Japan; 6 Department of Obstetrics and Gynecology, Mie University Graduate School of Medicine, Mie, Japan; 7 Department of Obstetrics and Gynecology, Showa University Northern Yokohama Hospital, Kanagawa, Japan; 8 Department of Obstetrics and Gynecology, Juntendo University, Tokyo, Japan; 9 Department of Health Services Research, Faculty of Medicine, University of Tsukuba, Ibaraki, Japan; 10 Department of Obstetrics and Gynecology, Nippon Medical School, Tokyo, Japan; 11 Department of Obstetrics and Gynecology, Fukushima Medical University, Fukushima, Japan; 12 Maeda Clinic, Shizuoka, Japan; 13 Department of Obstetrics and Gynecology, University of Nagasaki, Nagasaki, Japan

**Keywords:** Access to healthcare, Nightshift, Intensification, Concentration, Japan, Cerebral palsy

## Abstract

**Objective:**

This study estimated the effects of weekend and off-hour childbirth and the size of perinatal medical care center on the incidence of cerebral palsy.

**Methods:**

The cases were all children with severe cerebral palsy born in Japan from 2009 to 2012 whose data were stored at the Japan Obstetric Compensation System for Cerebral Palsy database, a nationally representative database. The inclusion criteria were the following: neonates born between January 2009 and December 2012 who had a birth weight of at least 2000 g and gestational age of at least 33 weeks and who had severe disability resulting from cerebral palsy independent of congenital causes or factors during the neonatal period or thereafter. Study participants were restricted to singletons and controls without report of death, scheduled cesarean section, or ambulance transportation. The controls were newborns, randomly selected by year and type of delivery (normal spontaneous delivery without cesarean section and emergency cesarean section) using a 1:10 case to control ratio sampled from the nationwide Japan Society of Obstetrics and Gynecology database.

**Results:**

A total of 90 cerebral palsy cases and 900 controls having normal spontaneous delivery without cesarean section were selected, as were 92 cerebral palsy cases and 920 controls with emergent cesarean section. A significantly higher risk for cerebral palsy was found among cases that underwent emergent cesarean section on weekends (odds ratio [OR] 1.72, 95% confidence interval [CI] 1.06–2.81) and during the night shift (OR 2.29, 95% CI 1.30–4.02). No significant risk was found among normal spontaneous deliveries on weekends (OR 1.63, 95% CI 0.97–2.73) or during the quasi-night shift (OR 1.26, 95% CI 0.70–2.27). Regional perinatal care centers showed significantly higher risk for cerebral palsy in both emergent cesarean section (OR 2.35, 95% CI 1.47–3.77) and normal spontaneous delivery (OR 2.92, 95% CI 1.76–4.84).

**Conclusion:**

Labor on weekends, during the night shift, and at regional perinatal medical care centers was associated with significantly elevated risk for cerebral palsy in emergency cesarean section.

## Key message

Labor on weekends, during the night shift, and at regional perinatal medical care center was associated with significantly elevated risk for cerebral palsy.

## Introduction

Over the past two decades, a growing body of evidence has demonstrated an association between work outside of the Monday through Friday schedule and increased morbidity and mortality in hospital settings [1]. Most studies that have found worsened outcomes in patients admitted during the weekend and during off-hour shifts have evaluated patients requiring urgent or emergency intervention, with conditions such as stroke [2], myocardial infarction [3], traumatic brain injury [4], pulmonary embolism [5], gastrointestinal hemorrhage [6], pediatric surgery [7], and mortality in critical care admission [8]. In obstetrical and perinatal outcomes, several studies have indicated the unfavorable influence of weekend and night delivery on neonatal death [9–14], birth-related injury,[14, 15] asphyxia, [16] intraventricular hemorrhage, [11] neonatal encephalopathy [17], maternal complication [18], and perinatal complication [12, 14, 18, 19]. Recent studies have analyzed the impact of staff fatigue [20], lack of medical personnel [17, 18], high volume [21], and institutional characteristics (e.g., academic teaching hospital [22] or secondary/tertiary hospital [23, 24]) on weekend and off-hour outcomes. A report from Sweden found an increased risk of asphyxia in infants born at night [16]. This indicates a possible negative weekend and off-hour effect on obstetrical care. Definitive conclusions have not been reached in this area due to narrowly focused research settings and the range of disease and symptom outcomes assessed [11, 18, 21, 25–28].

This study focuses on cerebral palsy (CP) as an outcome to evaluate weekend and off-hour effects in Japanese settings. In Japanese obstetrical care, stillbirth outcomes have improved, but risk for CP remains among surviving infants [29]. One study that used data from one hospital in Japan reported that adverse neonatal outcomes among cesarean births increased during the night [30]. The number of obstetricians is decreasing in Japan [31], and access to obstetric care is being reduced, especially on weekends and during off-hours. To tackle this urgent and problematic shortage, perinatal medical care has been consolidated, and tertiary (comprehensive and regional) perinatal care centers have been established. This consolidation may modify weekend and off-hour effects. This study evaluated whether there are weekend and off-hour effects and examined the potential effects of the type of perinatal medical care center on the incidence of CP, using nationwide Japanese databases.

## Methods

### Study design and participants

**Fig. 1:**
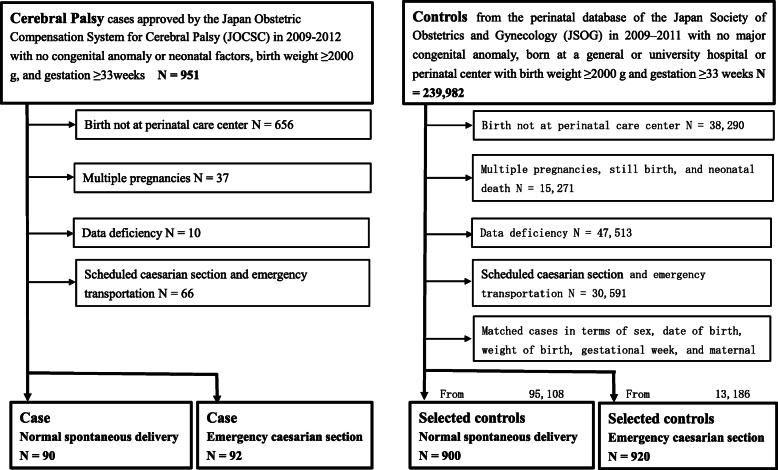
Study flow diagram

This was a retrospective case-control study that used data extracted from nationwide Japanese databases, namely, the Japan Obstetric Compensation System for Cerebral Palsy (JOCSC) and the Japan Society of Obstetrics and Gynecology (JSOG). The JOCSC is a government-supervised compensation system intended to provide prompt, no-fault compensation for children diagnosed with severe CP caused by trauma during labor and delivery and for their families, as well as to provide information that could help in prevention, the early resolution of disputes, and improving the quality of obstetric health care. All cases were approved for compensation by the JOCSC Operations Branch review board, which consists of obstetricians, pediatricians, midwives, and lawyers. Inclusion criteria for this study were the following: neonates born between January 2009 and December 2012, with a birth weight of at least 2000 grams and a gestational age of at least 33 weeks, and with a severe disability resulting from CP independent of congenital causes or factors during the neonatal period or thereafter. A comparable and compatible database period was used because the diagnosis of CP cases completed at 5 years old in JOCSC criteria and the data format of JSOG database was changed in 2013. In CP cases, disability is certified as having a first- or second-degree severity, according to the grade of disability definitions in the Welfare of Physically Disabled Persons Act [32], equivalent to grade three or severe in the gross motor function classification system. The study flow diagram is shown in Fig. 1.

Control group data were obtained from the JSOG perinatal database, an offline national and peerless birth registry that was established in 1974. The JSOG database contains attending physician-submitted annual data, in a standardized common format, for all pregnant women treated at the 253 comprehensive and regional perinatal care centers of the Perinatal Research Network of Japan [33]. Both types of care centers accept high-risk pregnant women and newborns with serious conditions, and both offer advanced perinatal care. Comprehensive perinatal care centers have more staff and equipment for special care and accept round-the-clock emergency referrals [34]. Data are stored with strict quality control implemented by the JSOG Perinatal Committee. The JSOG database, described in detail previously [35], is the largest obstetrics database in Japan, representing about 10% of the total number of births throughout the country each year. The JSOG database was used, despite its lack of comprehensive hospital and clinic coverage, because no comparable database exists in Japan.

Generally, in case-control studies, case and control groups should be sampled from a common population in terms of causal CP relationships, dates, and times. Controls sampled from the JSOG included birth records from comprehensive and regional perinatal care centers to match the backgrounds of the cases. Births following transportation by ambulance from other facilities incorporate challenging backgrounds. Therefore, study participants were restricted to cases and controls from perinatal care center hospitals without ambulance transportation for whom complete data for all analyzed items were available in the JOCSC database (for those born 2009–2012) in March 2017 and in the JSOG database (for those born in 2009–2011). To avoid the inclusion of CP cases among the controls, controls who matched cases in terms of sex, date of birth, birth weight, gestational week, and maternal age were excluded. Cases and controls with scheduled cesarean sections were excluded because scheduled procedures typically do not occur on the weekend or during a night shift. Instrumental delivery was included in the normal (vaginal) spontaneous delivery category. Because obstetrical processes for normal spontaneous delivery and emergence cesarean were identical, we applied stratified matching and stratified analysis for normal spontaneous delivery and delivery with emergent cesarean section. Matched analysis in matched sampled case-control studies can induce sampling bias [36], so we conducted un-matched analyses. Case and control data were extracted from the delivery medical records.

In case-control studies, matching is a means of providing a more efficient stratified analysis rather than a direct means of preventing confounding [36]. The data in the JSOG contained a few outlier values, typographical errors, and transfer errors from the hospital to the JSOG. To reduce the possibility of including inappropriate controls and to increase statistical power, cases were matched with 10 controls by birth year (2009, 2010, and 2011–2012) and type of birth (normal spontaneous delivery without cesarean section and emergency cesarean section).

### Assessments

The weekend was defined as Saturdays, Sundays, national holidays, and December 29–January 3. All other days were defined as weekdays. For the definition of off-hour time shifts, we aggregated blocks of time into three shifts: day (0800–1800), quasi-night (1800–2400), and night (2400–0800). The definition of pregnancy-induced hypertension (PIH) included preeclampsia and gestational hypertension. Gestational hypertension was defined as onset of hypertension after 20 weeks’ gestation (defined as systolic blood pressure of 140 mmHg or diastolic blood pressure of 90 mmHg) on at least two occasions, 4 h apart. Preeclampsia was defined as gestational hypertension with proteinuria (0.3 g in a 24-h urine specimen or a protein-to-creatinine ratio of > 0.30). Preterm labor is labor that begins before the 37th week of pregnancy. Fetal growth restriction was assessed using the standard deviation (SD) for birth weight [37].

### Statistical analyses

Continuous variables were reported as means ± SDs and were compared using Welch’s *t* test. Categorical variables were reported as frequencies and proportions and were compared using a chi-squared test. The relationships between CP and weekend/off-hour delivery were evaluated using multivariable logistic regression with CP as the dependent variable. The independent variables in Model 1 for cases and controls without cesarean section included day, perinatal care center level (comprehensive or regional ), shift, and year of delivery; Model 2 added the covariates prescription of prostaglandin or oxytocin, maternal age, parity, PIH, preterm labor, gestational week, birth weight, and SD for birth weight. Models 3 and 4 included the same covariates as Models 1 and 2 for cases and controls with normal spontaneous delivery. Models 5 and 6 included the same covariates as Models 1 and 2 for cases and controls with emergency cesarean section. The results were expressed as an odds ratios (ORs) and 95% confidence intervals (CIs). The goodness of fit of the multivariate models was evaluated using the Hosmer–Lemeshow test. Two-tailed *P* values less than 0.05 were used to define statistical significance. All analyses were conducted using Stata version 13.0 (STATA Corporation, College Station, TX, USA).

## Results

A total of 182 CP cases with normal spontaneous delivery without cesarean section and with emergent cesarean section (Table 1) were selected by year (2009, 64 [35.2%]; 2010, 49 [26.9%]; 2011–2012, 69 [37.9%]) from the JOCSC database (Table 1). These were matched to 1820 controls, also sampled by year (2009, 640 [35.2%]; 2010, 490 [26.9%]; 2011–2012, 690 [37.9%]). In all cases of either normal spontaneous delivery or emergent cesarean section, significantly more preterm labor (*P* < 0.001), fewer gestational weeks (*P* < 0.001), more fetus growth restriction (SD for birth weight; *P* < 0.001), more weekend delivery (*P* = 0.015), and more use of regional perinatal care centers (*P <* 0.001) were seen in the CP cases.

**Table 1:** Demographics and prenatal participant characteristics

Variable	CP (*N* = 182)	Control (*N* = 1820)	*P* value
Birth year			
2009	64 (35.2%)	640 (35.2%)	NA
2010	49 (26.9%)	490 (26.9%)	
2011–2012	69 (37.9%)	690 (37.9%)	
Delivery			
Normal	90 (49.5%)	900 (49.5%)	NA
Emergency CS	92 (50.5%)	920 (50.5%)	
Age, years	32.0 ± 5.1	31.9 ± 5.4	0.865
Parity			
One or more	77 (42.3%)	688 (37.8%)	0.233^a^
Prescription			
Prostaglandin	22 (12.1%)	156 (8.6%)	0.112^a^
Oxytocin	41 (22.5%)	479 (26.3%)	0.266^a^
PIH	13 (7.1%)	116 (6.4%)	0.687^a^
Preterm labor	68 (37.4%)	230 (12.6%)	< 0.001^a^
Gestational week	37.8 ± 2.3	38.8 ± 1.7	< 0.001
SD for birth weight	-0.304 ± 1.032	0.003 ± 1.039	< 0.001
Perinatal care centers			
Comprehensive	60 (33.0%)	992 (54.5%)	< 0.001^a^
Regional	122 (67.0%)	828 (45.5%)	
Day			
Weekday	122 (67.0%)	1344 (73.9%)	0.015^a^
Weekend	60 (33.0%)	476 (26.2%)	
Time shift			
Day	89 (48.9%)	1015 (55.4%)	0.088^a^
Quasi-night	44 (24.2%)	435 (23.9%)	
Night	49 (26.9%)	370 (20.3%)	

Data are expressed as means ± SDs or numbers (percentages). *P* values are derived from Welch’s *t* test

*NA* not applicable or being used in matching, *CP* cerebral palsy, *PIH* pregnancy-induced hypertension, *CS* caesarian section, *SD* standard deviation

^a^The chi-squared test

Table 2 shows that significantly more cases than controls had night deliveries, adjusted for year and obstetric condition (Model 2: OR 1.56, CI 1.05–2.32). Significantly more cases than controls also had weekend deliveries, adjusted for year and obstetric condition (Model 2: OR 1.59, CI 1.12–2.25). In addition, significantly more cases than controls had delivery in regional perinatal care centers, adjusted for year (Model 1: OR 2.41, CI 1.74–3.33) and obstetric condition (Model 2: OR 2.50, CI 1.78–3.50).

**Table 2:** Odds ratios for weekend and off-hour effects on cerebral palsy

Variable	Bivariate	Model 1	Model 2
Perinatal care centers			
Comprehensive	1.00	1.00	1.00
Regional	2.40, 1.74–3.32	2.41, 1.74–3.33	2.50, 1.78–3.50
Day			
Weekday	1.00	1.00	1.00
Weekend	1.50, 1.08–2.08	1.41, 1.01–1.97	1.59, 1.12–2.25
Shift			
Day	1.00	1.00	1.00
Quasi-night	1.25, 0.86–1.83	1.28, 0.87–1.87	1.29, 0.87–1.92
Night	1.48, 1.02–2.14	1.43, 0.98–2.08	1.56, 1.05–2.32
Hosmer–Lemeshow test		*P* = 0.636	*P* = 0.415

Data are expressed as odds ratios and 95% confidence intervals with logistic regression models

Cases: *N* = 182, controls: *N* = 1820; covariates: (Model 1) birth year; (Model 2) adding birth year, prescription of prostaglandin, prescription of oxytocin, maternal age, parity, pregnancy-induced hypertension, preterm labor, gestational week, standard deviation for birth weight, and delivery

For those with normal spontaneous delivery (Table 3), significantly more cases than controls were delivered at regional perinatal care centers, adjusted for year (Model 3: OR 2.69, CI 1.69–4.30) and obstetric condition (Model 4: OR 2.92, CI 1.76–4.84).

**Table 3:** Odds ratios for weekend and off-hour effects on cerebral palsy for normal spontaneous delivery

Variable	Bivariate	Model 1	Model 2
Perinatal care centers			
Comprehensive	1.00	1.00	1.00
Regional	2.65, 1.66–4.21	2.69, 1.69–4.30	2.92, 1.76–4.84
Day			
Weekday	1.00	1.00	1.00
Weekend	1.20, 0.76–1.91	1.17, 0.73–1.88	1.63, 0.97–2.73
Shift			
Day	1.00	1.00	1.00
Quasi-night	1.44, 0.85–2.44	1.51, 0.89–2.58	1.65, 0.93–2.95
Night	1.04, 0.62–1.75	1.00, 0.59–1.71	1.26, 0.70–2.27
Hosmer–Lemeshow test		*P* = 0.075	*P* = 0.897

Data are expressed as odds ratios and 95% confidence intervals with logistic regression models

Cases: *N* = 90, controls: *N* = 900; covariates: (Model 3) birth year; (Model 4) adding birth year, prescription of prostaglandin, prescription of oxytocin, maternal age, parity, pregnancy-induced hypertension, preterm labor, gestational week, and standard deviation for birth weight

For those with cesarean section (Table 4), significantly more cases than controls had weekend (OR 1.75, CI 1.09–2.80) or night (OR 2.40, CI 1.40–4.11) deliveries (Model 5). In the multivariable model adding obstetric conditions (Model 6), cases again had significantly more weekend (OR 1.72, CI 1.06–2.81) and night (OR 2.29, CI 1.30–4.02) deliveries. Significantly more cases than controls had a cesarean section in a regional perinatal care center, adjusted for year (Model 5: OR 2.24, CI 1.42–3.53) and obstetric condition (Model 6: OR 2.35, CI 1.47–3.77).

**Table 4:** Odds ratios for weekend and off-hour effects on cerebral palsy for delivery with emergent caesarian section

Variable	Bivariate	Model 1	Model 2
Perinatal care centers			
Comprehensive	1.00	1.00	1.00
Regional	2.19, 1.40–3.43	2.24, 1.42–3.53	2.35, 1.47–3.77
Day			
Weekday	1.00	1.00	1.00
Weekend	1.89, 1.19–2.99	1.75, 1.09–2.80	1.72, 1.06–2.81
Shift			
Day	1.00	1.00	1.00
Quasi-night	1.06, 0.61–1.84	1.04, 0.59–1.81	1.15, 0.64–2.07
Night	2.46, 1.45–4.17	2.40, 1.40–4.11	2.29, 1.30–4.02
Hosmer–Lemeshow test		*P* = 0.700	*P* = 0.561

Data are expressed as odds ratios and 95% confidence intervals with logistic regression models

Cases: *N* = 92, controls: *N* = 920; covariates: (Model 5) birth year; (Model 6) adding birth year, prescription of prostaglandin, that of oxytocin, maternal age, parity, pregnancy-induced hypertension, preterm labor, gestational week, and standard deviation for birth weight

The Hosmer–Lemeshow test showed a fairly good fit for all models (no significant *P* values were observed in any models).

## Discussion

Using two nationwide perinatal databases, our case-control study in comprehensive and regional perinatal care centers found no weekend or off-hours effects on CP evident in normal spontaneous deliveries. However, we observed a 1.7-fold weekend and 2.3-fold night on CP for delivery with emergency cesarean section without emergency transportation. To the best of our knowledge, this is the first study to investigate the risk for CP associated with delivery during the weekend and off-hour periods.

In Japan, approximately 2500 facilities provide delivery services for approximately one million deliveries per year [31]. Because pregnant women often prioritize the accessibility and comfort of delivery facilities, deliveries are not intensively performed, and more than half of all deliveries occur in private clinics operated by one or two obstetricians [31]. Nevertheless, the perinatal mortality rate and maternal mortality rate in Japan in 2012 were 4 per 1000 and 4 per 100,000 births, respectively [38], the lowest figures worldwide. Thus, the safety standards of prenatal practice in Japan are at least equivalent to those in other developed countries.

The incidence of CP in Japan is estimated to be 2.0 per 1000 births [39], which is about the same as that in other developed countries [40]. CP occurrence related to labor management should be limited, but the current study indicates that the risk might be elevated on the weekend and at night even in comprehensive and regional perinatal care centers that offer advanced perinatal care.

Previous studies on weekend and off-hour effects on obstetrical and perinatal outcomes have proposed several explanations: staff fatigue [20], a lack of medical personnel [17, 18], high volumes [21], and institutional characteristics [22–24]. Other previous studies have reported no difference in patient management on the weekend versus on weekdays for spontaneous labor [41] nor any increase in adverse maternal and neonatal outcomes from cesarean births at night [25]. Another study of weekend and off-hour effects on other health outcomes have offered explanations, such as fewer personnel, lower staffing levels, reduced numbers of senior staff, cross-cover of clinical specialties, proportionally more urgent procedures, deficits in continuity of care, and presenting patients often being in a more complex or more critical condition than those who typically present on weekdays [42]. Our target hospitals, comprehensive and regional perinatal care centers, must meet staff and facility requirements for 24 h operation; however, medical staff is reduced during the night and on weekends, meaning that fewer obstetricians are available to handle serious cases. Weekend and night CP effects during emergency cesarean sections might be the result of multiple simultaneous factors: lower staffing levels, fewer senior staff, cross-cover of clinical specialties, and proportionally more urgent procedures on weekends and at night.

In all models, regional perinatal care centers had a 2.2-fold or higher risk of CP compared to comprehensive perinatal care centers. Generally, the minimum staffing and equipment requirements at regional perinatal care centers are lower than for comprehensive perinatal care centers [43]. Severe institutional risk conditions induced by deliveries may thus occur more frequently in regional perinatal care centers.

In a previous study, delays in diagnosis and treatment, including maternal transport, intervention, and blood transfusion, were associated with maternal death in Japan [26]. In fact, such cases encounter several problems in diagnostic procedures, treatment strategies, and inter- and intra-hospital systems [31]. In 2000, inadequate obstetric services were associated with maternal mortality in Japan. Reducing the number of deliveries in single-obstetrician facilities and establishing regional 24 h inpatient obstetric facilities for high-risk cases might reduce maternal mortality in Japan [44].

This weekend and holiday problem in regional perinatal care centers may reflect the current condition of obstetric medical care, in particular, the shortage of obstetricians. To tackle this shortage, the Medical Reform Committee of JSOG published its Grand Design 2015 [45], which recommended that obstetric services be consolidated in secondary and tertiary care hospitals, and regional and comprehensive perinatal care centers, to reach a sufficient number of full-time obstetricians. Consolidation of obstetric facilities impairs access, but a higher caseload and better staffing have the potential advantages of better clinical outcomes and reduced costs [46]. Metropolitan areas could develop plans to consolidate perinatal medical care; however, rural regions would not be able to achieve similar consolidation. The redistribution of care centers and obstetricians should also be accelerated. Comprehensive perinatal care centers are not distributed throughout the country to provide efficient access to perinatal care. According to the current results, appropriate management and intensive care is needed in regional perinatal care centers during the night and on weekends and holidays. Consolidation progressed during the study period 2009–2011. Further study is needed to fully capture the current situation.

This study has several strengths. First, we included a large number of CP patients from a nationwide database. Statistical power was estimated at 86.0, 62.6, and 97.8%, respectively, for night, weekend, and regional perinatal care center effects for the bivariate analysis of delivery with emergent cesarean section. Second, data were drawn from available medical information and did not include self-administrated questionnaires, attenuating potential recall bias. However, this study also had several limitations. First, to provide grounds for comparison, it focused on cases and controls in comprehensive and regional perinatal care centers without ambulance transportation. Labor in general obstetrics clinics and hospitals with a limited number of obstetricians and midwives might be associated with exaggerated risk during weekend and off-hour delivery or in emergent cases with ambulance transportation. Second, only participants with births at 33 or more weeks were eligible to participate, so night and weekend effects were not studied for births outside of these restrictions. Third, the quality of the dataset was not balanced because the JOCSC data were manually confirmed by JOCSC investigators but the JSOG data were automatically transcribed from electronic patient records. Individual risk behaviors, i.e., alcohol drinking and drug use, were assessed with different questions between databases. No pair of comparable databases exists. Individual risk behaviors occur more often during weekends and at night and may therefore be a cause of weekend and night effects produced in this study [2]. Pregnant women usually avoid risk behaviors; however, the potentially confounding effects of individual behaviors were not analyzed in the current study. Fourth, the time between amniotic rupture and total delivery was not included in the analysis due to uncertain data recording.

## Conclusions

In conclusion, in a case-control analysis, we found that weekend and night delivery were significantly associated with an elevated risk of CP in emergency cesarean sections without ambulance transportation in comprehensive and regional perinatal care centers. Regional perinatal care centers exhibited significantly higher risk for CP than comprehensive perinatal care centers. These findings should motivate health policymakers to consolidate perinatal medical care and to ensure that management and intensive care are appropriately applied in regional perinatal care centers during the night as well as on weekends and holidays.

## Data Availability

The data used to derive our conclusions are unsuitable for public deposition due to ethical restrictions imposed by the institutional ethics committee, as the data contain sensitive information on participants and facilities.

## Abbreviations

CI: Confidence interval
CP: Cerebral palsy
JOCSC: The Japan Obstetric Compensation System for Cerebral Palsy
JSOG: The Japan Society of Obstetrics and Gynecology
OR: Odds ratio
PIH: Pregnancy-induced hypertension
SD: Standard deviation
